# The Relationship Between the Three Models of Emotional Intelligence and Psychopathy: A Systematic Review

**DOI:** 10.3389/fpsyt.2018.00307

**Published:** 2018-07-12

**Authors:** Raquel Gómez-Leal, María J. Gutiérrez-Cobo, Rosario Cabello, Alberto Megías, Pablo Fernández-Berrocal

**Affiliations:** ^1^Department of Basic Psychology, Faculty of Psychology, University of Málaga, Málaga, Spain; ^2^Department of Developmental and Educational Psychology, University of Granada, Granada, Spain

**Keywords:** emotional intelligence, psychopathy, performance-based ability models, self-report ability models, self-report mixed models

## Abstract

Given the many instances of violence and crime that occur as a consequence of psychopathy, it is vital to find those variables that can reduce the expression of such behaviors. In this regard, one potentially useful variable is that known as Emotional Intelligence (EI) or the ability to perceive, use, understand, and regulate emotions. EI has been categorized according to three main approaches: performance-based ability, self-report ability, and self-report mixed models. Given the emotional deficits of the psychopathic population, EI could be a protective factor. Several studies have analyzed the relationship between EI and psychopathy, but the results are unclear. This disparity may be due to the EI model employed to measure EI. The aim of our research is to systematically review the relationship between the different models of EI and psychopathy, both in the total and clinical/inmate sample. We searched Scopus, Pubmed, and PsicINFO to find relevant articles. Twenty-nine eligible studies were found. They were divided according to the model of EI and the sample used. The results for the total sample differ according to the measure of EI: when the performance-based ability model is used, the majority of studies find a negative relationship between EI and psychopathy. When using self-reports, the results are inconsistent. The findings with the clinical/inmate sample are in the same direction as the total sample. In conclusion, the results suggest that higher EI abilities measured through performance-based ability models—but not through self-reports—are related to lower psychopathy deficits. Limitations and clinical implications are discussed.

## Introduction

Emotions are very important in our lives; every day we feel many emotions that allow us to better adapt to the world around us. We use our emotions in multiple contexts, including social and friendly interactions, dealing with the moments leading up to a stressful event, and when attempting to understand and help others with their problems. Our emotions have a great impact on our psychological wellbeing. Emotional intelligence (EI) is a recently developed concept that encompasses a set of emotional aptitudes, which was first presented in 1990 by Peter Salovey and John Mayer. These authors [(1), p. 10] have defined this construct as:

*The ability to perceive accurately, appraise, and express emotion; the ability to access and/or generate feelings when they facilitate thought; the ability to understand emotion and emotional knowledge; and the ability to regulate emotions to promote emotional and intellectual growth*.

Since the emergence of the concept of EI, multiple methods of evaluation have appeared to measure this construct. However, not all of these instruments define EI in the same way or measure it using the same evaluation methods. Therefore, there is a need to organize the literature according to these methods. For this purpose, Joseph and Newman (2) suggested that EI can be divided into three models, depending on the kind of instruments and theoretical framework employed. These models are the *performance-based ability model*, the *self-report ability model* and the *self-report mixed model*.

The performance-based ability model regards EI as a skill focused on processing emotional information in a way that unifies emotions and reasoning (1). This model understands EI as a form of intelligence based on a set of emotional aptitudes (3), and employs performance tests where participants are required to solve emotional problems in which there are correct and incorrect answers. The Mayer-Salovey-Caruso Emotional Intelligence Test [MSCEIT; (4)] is the most important test of this EI model (5). The Self-Report ability model, like the performance-based ability model, conceptualizes EI as a set of emotional aptitudes, but uses self-reports to measure the construct, where participants report their subjective beliefs about their own EI and where there are no correct and incorrect responses. The Trait Meta-Mood Scale [TMMS; (6)] is the best-known test for this model (7, 8). Finally, the self-report mixed model also measures EI with self-report instruments, and it includes, in the definition of the construct, personality factors, mental abilities and motivation. The most representative tests of this model are the Bar-on Emotional Quotient Inventory [EQ-i; (9)] and The Trait Emotional Intelligence Questionnaire [TEIQue; (10)].

Recent literature has attempted to seek multiple relationships between EI and relevant psychological dimensions (11–13). In particular, people who are able to perceive, know, and manage their emotions are usually better able to handle emotional problems and thus have lower psychological burdens (14). EI is also positively related to life satisfaction and happiness (15). On the other hand, negative relationships have been found between EI and the dimensions of psychopathology. For example, Ahmadpanah et al. (16) showed that higher levels of EI were associated with lower levels of anxiety in a sample of students. In addition, Jahangard et al. (17) demonstrated that EI training in depression and borderline personality disorder patients produced a decrease in depressive symptoms. Moreover, EI has also been negatively related to the Dark Triad (15, 18).

The Dark Triad (DT) consists of three distinct personality traits: machiavellianism, narcissism, and psychopathy. These three traits represent behavioral tendencies toward deception, self-promotion, and aggressiveness (19). Recently, attention has been paid to the study of the DT, with a number of studies analysing its relationship with other constructs. For instance, a positive correlation has been found between DT and mental toughness, physical activity, intolerance of uncertainty, sleep disturbances, and sensitivity to anxiety in young adults (20, 21).

Machiavellianism is defined by manipulative behaviors, insincerity, and callousness (22) while narcissism is characterized by dominance, exhibitionism and exploitation as well as feelings of superiority (23). Finally, psychopathy is characterized by impulsive, thrill-seeking behaviors combined with anxiety, dishonesty, egocentricity, manipulation, and exploitation of others (24). It has been demonstrated that these three constructs are readily distinguishable from each other (19). However, research has shown that they share a callous and antagonistic core dimensions (25). Moreover, it has been suggested that there is a high degree of overlap between the nomological networks of psychopathy and machiavellianism (26).

Psychopathy traits have been associated with low empathy, and psychopathic individuals often repeat the damage done to their victims on several occasions, proving that they are not capable of generating empathy in response to others (27). Further, some researchers have shown that individuals with psychopathic traits are deficient in recognizing emotional facial expressions (28). Given that EI is closely related to these deficits, our proposal is to analyse the possible relationship between EI and psychopathy.

As with the case of EI, psychopathy is measured by different questionnaires that contain various scales that measure different constructs, since psychopathy is not defined in the Diagnostic and Statistical Manual of Mental Disorders [5th ed; DSM-V; (29)] as such. Each measurement instrument has an operational definition of the integrated construct within a theoretical and conceptual framework. The results of several studies (30–32) have indicated that the majority of psychopathy measures are weakly intercorrelated. For example, the two most studied measures of adult psychopathy (PCL-R and PPI) identify overlapping, but rather distinct, constructs (33). On the one hand, Hare's Psychopathy Checklist [PCL, (34); PCLR, (23, 35)] includes many items addressing criminal and antisocial behavior. In particular, PCL Factor 1 [i.e., “Interpersonal-Affective” scale; (36)] is uniquely distinguished by superficial charm, a deceitful interpersonal style, a lack of empathy, and shallow affect (It is composed of elements consistent with the traditional conceptualizations of psychopathy). In contrast, PCL Factor 2 [i.e.,“Social Deviance” scale; (35)] is characterized by general impulsivity, irresponsibility, and past criminal and antisocial behavior (not specific to psychopathy). However, if psychopathy is evaluated with the Psychopathic Personality Inventory-Revised [PPI-R; (36)], this excludes items that refer to criminal or antisocial behavior, so that criminality does not appear to be the central feature of psychopathy (37). The PPI is also composed of two factors: Factor 1 (“Fearless Dominance”), that reflects the more interpersonal-affective features of the disorder, and Factor 2 (“Self-Centered Impulsivity or Impulsive Antisociality”) that reflects the more impulsive-behavioral features of the disorder. This personality-based approach is congruent with the conceptualization of psychopathy as a personality disorder. The two measures explain >30% of each other's variance (38). Specifically, the PCL-R's Interpersonal-Affective factor is weakly associated with the PPI's Fearless Dominance (*r* = 0.21), and Self-Centered Impulsivity (*r* = 0.20) factors. In contrast, the PCL-R's Social Deviance factor is weakly associated with the PPI's Fearless Dominance (*r* = 0.15) (39). Apart from these differences between these two main questionnaires, there are other scales also employed in the literature that we will define later in the method section [e.g., Self-Report Psychopathy Scale-III (SRP-III); (40)].

Although still not straightforward (37, 41), it appears that some psychopathy factors are related to violence (42). For instance, the Social Deviance factor of the PCL-R is a good predictor of violence. In addition, it appears that psychopaths often lie for reasons such as escaping punishment, earning money or even having sex (43). Moreover, some psychopaths maintain control over others by deception and manipulation, using an attractive personality (44). Increasingly, the psychopathic construct is used as a predictor of danger (45–47). In fact, psychopathic traits have been shown to be valid predictors of outcomes such as delinquency or aggression (48–50). Delinquents with psychopathy begin to commit delinquent acts at an early age (51). In addition, psychopaths commit a considerably wider range of crimes than non-psychopaths (35, 52). Moreover, it is important to emphasize that psychopaths have a higher risk of recidivism than non-psychopaths, a fact that can be seen in a meta-analysis performed by Hemphill et al. (53) where it is shown that they are three times more likely to commit crime, and four times more likely to commit a violent offense. It has also been demonstrated (54) that emotional regulation is negatively related to psychopathy in children and adolescents.

It is expected that, given their emotional alterations, psychopaths present a deficit in EI. By analysing the studies that describe the relationship between EI and psychopathy, we can observe that the results are inconclusive. In particular, while some studies have found a negative relationship between EI and psychopathy (55, 56), others have found a positive relationship (57), and some have found no relationship at all (58). Given the variability in the instruments used to measure EI, this discrepancy between the findings of different studies could be due to the use of an objective or subjective measure of EI (59, 60). This possibility prompted the present study. In particular, the purpose of this work is to review the literature on the relationship between the three models of EI and psychopathy, with the specific aim of analysing how each EI model relates to psychopathy in order to determine which of the models is the most predictive. In addition, a second objective is to choose those studies that employ a clinical or inmate sample (they had been in prison, committed crimes, or had been admitted to a psychiatric center), with the idea of analysing this sample in isolation.

We expect to primarily find a negative correlation between EI and psychopathy when a performance-based model is used, and when using self-report measures, we expect the results to be more inconsistent given that there are studies (60) demonstrating that not all EI models predict certain outcomes in the same way. For instance, in a study by Gutiérrez-Cobo et al. (60) EI measured through the performance-based ability model predicted better performance in emotional cognitive tasks than the models using self-reports.

## Materials and methods

### Literature search and inclusion criteria

Medline, Scopus and PsycINFO databases were carefully searched to find suitable articles that were available until June 2017. The terms were introduced as follows: (a) “emotional intelligence” and “psychopath” (b) “emotional intelligence” and “psychopathic” (c) “emotional intelligence” and “psychopathy”. These combinations must appear in the title, abstract or keywords. Finally, the inclusion criteria were articles written in English or Spanish, as well as those articles including measures of any EI model (performance-based ability test, a self-report mixed model, or a self-report ability model), and psychopathic traits. Exclusion criteria were: unpublished research, comments, editorials, master's theses, or dissertations, and non-English or non-Spanish language publications.

**Figure 1:**
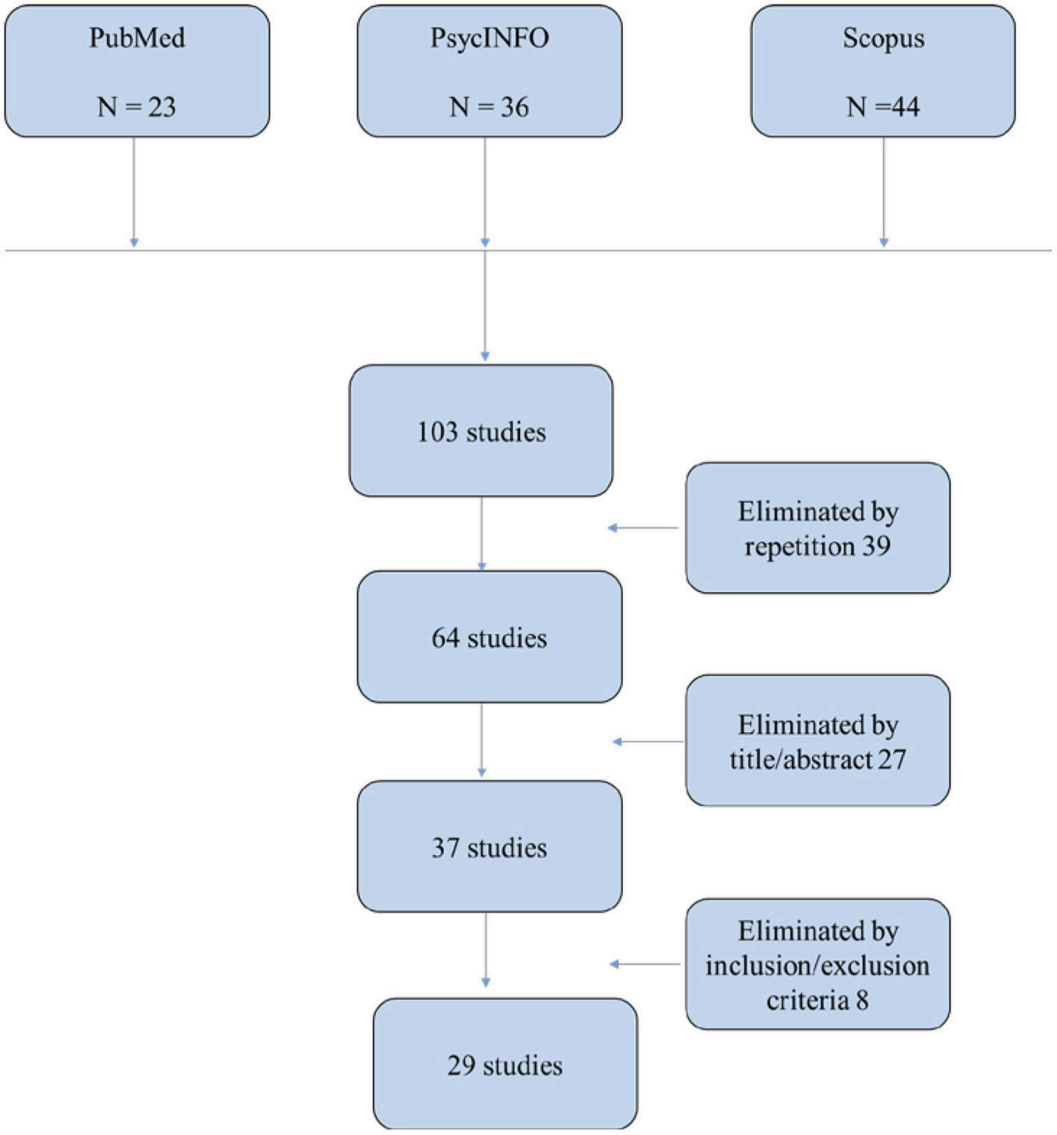
Prisma Flow-diagram for literature included in this study [see (61)].

We identified a total of 103 references. After removing duplicates, this resulted in 64 studies. Two reviewers independently assessed the titles and abstracts of all of the reports identified. Of these 64 studies, only 37 were selected to review the full text taking into account the inclusion/exclusion criteria specified, 29 studies were finally included. Disagreements were resolved by deliberation with the senior reviewer. The process of finding and selecting the items is shown in Figure 1.

### EI instruments

We next define the instruments used to measure EI in the studies included in this review, divided by model.

#### Performance-based ability models

Mayer-Salovey-Caruso Emotional Intelligence Test [MSCEIT; (4)] is a 141-item test divided into two areas: experiential, which is composed of the branches of perceiving emotions and facilitating thought, and strategic, composed of the branches of understanding emotions and managing emotions. Each branch is composed of two separate tasks (62). These branches measure the ability to perceive, facilitate, understand, and manage emotions. Although there is no single correct answer, some answers are better than others. The psychometric properties of MSCEIT are adequate, since this test has a high reliability index (0.91) for both general and expert consensus (0.93) (62) and its test-retest reliability, for a time interval of 3 weeks, is 0.86 (63).

#### Self-report ability models

##### Trait-meta mood scale (TMMS)

Trait-Meta Mood Scale [TMMS; (6)] is a 30–item self-report. This test is divided into three scales: the attention to feeling scale (0.86) that evaluates the degree to which individuals think about or notice their feelings; the clarity of feeling scale (0.87) that assesses the extent to which individuals are able to identify, understand, and discriminate among their feelings, and the mood repair scale (0.82) which measures how well individuals regulate their moods and repair negative emotional experiences. Research has provided support for the reliability and validity of the TMMS as an index of EI (64).

##### Wong and law emotional intelligence scale (WLEIS)

Wong and Law Emotional Intelligence Scale [WLEIS; (65)] is a 16–item test. WLEIS is designed to assess an individual's self-perceived level of the ability to recognize and regulate his or her own emotions. Self-ratings are based on a 7–point scale anchored by “strongly disagree” on one end and “strongly agree” on the other. The internal consistency of the WLEIS was very good (0.86) (66).

##### Self-reported social skills inventory (SSI)

Self-reported Social kills Inventory [SSI: (67)] contains 90 items with six subscales that measure emotional expressivity, emotional sensitivity, emotional control, social expressivity, social Sensitivity, and social control. The SSI shows good test-retest reliability, ranging from 0.81 to 0.96 and has good internal consistency, with Cronbach's alpha coefficients ranging from 0.65 to 0.88 (67).

##### Schutte's self-report emotional intelligence scale (SEIS)

Schutte's Self-Report Emotional Intelligence Scale [SEIS; (68)] includes 33 items. It includes five items for emotional perception, six for emotional use, five for emotional understanding, and six for emotional management. According to Schutte et al. (68), it reports a reliability rating of 0.90. The EI total score is reliable for adults and adolescents; however, the utilizing emotions sub-scale has been shown to have poor reliability (69).

#### Self-report mixed models

##### The trait emotional intelligence questionnaire (TEIQue)

The Trait Emotional Intelligence Questionnaire [TEIQue; (10)]. Participants are asked to respond to 153 self-reflective items using a 7-point scale. It is composed of four factors: wellbeing, self-control, emotionality, and sociability. Internal consistencies of the facet and factor scores have been reported to range from 0.59 to 0.91, and from 0.85 to 0.91, respectively (70).

##### Trait emotional intelligence questionnaire—short form (TEIQue–SF)

Trait Emotional Intelligence Questionnaire—Short Form [TEIQue–SF; (71)] is a 30–item self-report scale, using a 7–point scale. This questionnaire evaluates four factors: wellbeing, self-control, emotionality and sociability, and has demonstrated adequate reliability and validity (71).

##### BAR-On EQ-i (EQ-i)

Bar-On EQ-i [EQ-i; (72)] consists of 133 items presented on a Likert rating scale. EQ-i has five subscales: intrapersonal, interpersonal, stress management, adaptability, and general mood, and total EQ. EQ-I has high internal consistency (0.97) on each of the five subscales and good test-retest reliability (0.79) (73, 74).

### Psychopathy scale

We next define the instruments used to measure psychopathy in the studies included in this review.

#### The psychopathy checklist—revised

(PCL-R)The Psychopathy Checklist—Revised [PCL-R; (35)] has 20 items, each of which is scored on a 3-point scale. Scores range from 0 to 40; the diagnostic cut off for psychopathy is 30 (24). The PCL-R has two factors, and these in turn are divided into two facets. Factor 1 (interpersonal-affective) is the facets that reflect a psychopath's affective deficits and interpersonal features, and in factor 2 (social deviance) subsumes the facets that reflect a psychopath's unstable lifestyle and antisocial behavior. The internal consistency of the total PCL-R score is acceptable (Cronbach's α 0.91).

#### Psychopathy checklist: youth version (PCL: YV)

Psychopathy Checklist: Youth Version [PCL: YV; (75)] is a 20–item rating scale for the assessment of psychopathic traits in male and female offenders aged 12–18. The questionnaire measures psychopathic characteristics for Interpersonal (Facet 1), Affective (Facet 2), Lifestyle (Facet 3), and Antisocial Behavior (Facet 4). The intra-class correlation coefficient (ICC 1, 1) = 0.90.

#### The psychopathic personality inventory revised (PPI-R)

The Psychopathic Personality Inventory Revised [PPI-R; (36)] consist of 154 items rated on a four-point scale. The PPI-R is divided into eight subscales comprising two factors: fearless dominance and impulsive antisociality (76). The scales of this test have good internal consistency (α = 0.78–0.92) and test–retest reliability (α = −0.82–0.95).

#### Self-report psychopathy Scale-III (SRP-III)

Self-Report Psychopathy Scale-III [SRP-III; (40, 77)] is a 64–item self-report scale. This test has a total score and a score for each of its 4 scales: interpersonal manipulation, callous affect, erratic lifestyle, and antisocial behavior. Research indicates the SRP-III has a satisfactory internal consistency [e.g., (78)].

#### The levenson self-report psychopathy scale (LSRP)

The Levenson Self-Report Psychopathy Scale [LSRP; (79)] has 26 items, with 16 items assessing primary psychopathy such as being selfish, uncaring, and manipulative, and 10 items evaluating secondary psychopathy including anti-social behavior, a self-defeating lifestyle and impulsivity. The LSRP is valid and reliable as it has 0.82 for primary psychopathy and 0.63 for secondary psychopathy.

#### Psychopathic personality inventory—short form (PPI-SF)

Psychopathic Personality Inventory—Short Form [PPI-SF; (80)] has 56 questions with 8 subscales: Machiavellianism, egocentricity, social potency, fearlessness, coldheartedness, impulsive nonconformity, blame externalization, carefree non-planfulness, and stress immunity.

#### Psychopathic personality inventory (PPI)

Psychopathic Personality Inventory [PPI; (80)] is a 187-item test that uses a 4-point scale anchored with “false” on one end and “true” on the other. The PPI consists of the following 8 subscales: stress immunity, social potency, fearlessness, impulsive nonconformity, blame externalization, Machiavellianism, egocentricity, and carefree non-planfulness. PPI has a good range of internal consistency from 0.78 to 0.87 in a student-and community sample and from 0.71 to 0.84 in an offender sample (80).

#### NEO psychopathy resemblance index (NEO PRI)

NEO Psychopathy Resemblance Index [NEO PRI; (81)] has 120 items and consists of four items for each of the 30 personality facets. Scores are combined to create an assessment of each of the Big Five personality dimensions: neuroticism, extraversion, openness to experience, conscientiousness, and agreeableness.

#### Self-report psychopathy scale (SRP)

Self-Report Psychopathy Scale [SRP; (82)] is a 64–item scale. The SRP produces a total score and four subscale scores: interpersonal manipulation, callous affect, and erratic and criminal tendencies. SRP has very good psychometric properties, as fully demonstrated in several studies [e.g., (77, 83, 84)].

#### Self-report psychopathy scale (SRP-4)

Self-Report Psychopathy Scale [SRP-4; (40)] has 64 items with four facets of psychopathy: antisocial behavior, interpersonal manipulation, cold affect, and impulsivity. The SRP-4 has good reliability (Cronbach's α = 0.81) (85).

#### Dark triad dirty dozen (DTDD)

Dark Triad Dirty Dozen [DTDD; (86)] measures narcissism (Cronbach's α = 0.85), Machiavellianism (Cronbach's α = 0.73), and psychopathy (Cronbach's α = 0.80) with four items each on a 7-point Likert-type scale.

#### Short dark triad (SD3).

Short Dark Triad [SD3; (87)] has 27 items. The SD3 measures narcissism (Cronbach's α = 0.72), Machiavellianism (Cronbach's α = 0.71), and psychopathy (Cronbach's α = 0.78).

## Results

Our investigation identified 29 studies that measured EI a total of 32 times, 11 of the 32 times in which EI was assessed, the study employed performance-based ability tests, while self-report ability tests were also used 10 times, and self-report mixed tests were used 11 times. Psychopathy was measured with 11 different scales in these 29 studies.

The results will be divided into two sections: in the first section, we will use all the articles found in our review, and thus the total sample will be included. In addition, we separate the articles according to the model of EI that they use (the self-report ability model, self-report mixed model, or performance-based ability model). In the second section, only the clinical/inmate sample will be included, understood as the one that has committed acts of aggression, been in jail or admitted to a psychiatric unit. As in the first section, the articles will also be divided according to the EI model used.

### Total sample

#### Performance-based ability model

We identified 11 studies where EI was measured through performance ability tests (Table 1). Eight of eleven studies found negative relationships between EI and psychopathy. One of the eleven studies found negative correlations for some scales, but positive correlations with perception ability. Finally, 2 of 11 studies found no relationship between EI and psychopathy. All these studies used MSCEIT as the EI measuring instrument.

**Table 1:** Studies using the Performance-based ability model.

**Study**	**EI scale**	**Psychopathy scale**	**Sample (N)**	**Principal results**
(55)	MSCEIT	PCL-R	374 male inmates	-Negative correlation between psychopathy and strategic EI. -Negative correlation between the Impulsive trait of psychopathy and both branches of strategic EI.
(88)	MSCEIT	PPI-R	188 male undergraduate students	-Modified PPI-R total scores inversely associated with MSCEIT, specifically, a deficit in understanding and managing emotions. -The impulsive antisociality scale correlated inversely with total, facilitating and understanding and managing emotions. - High anxious psychopathy had lower EI than low-psychopathy and low psychopathy comparison groups.
(89)	MSCEIT	PPI-R	55 employees of financial institutions (16 female)	-Negative correlation between total psychopathy and total EI. -Self-centered impulsivity was significantly negatively correlated with MSCEIT total scores, perceiving, facilitating and managing branches. -Coldheartedness was significantly negatively correlated with MSCEIT total scores and facilitating branch. - The interpersonal-affective scores were not significantly related to EI.
(90)	MSCEIT	SRP-III	162 undergraduate students (79 female)	- Psychopathy and its facets showed a significant negative association with MSCEIT.
(91)	MSCEIT	SRP-III	486 undergraduate students (254 female)	- Negative correlation between EI subscales and psychopathy and antisociability subscales.
(57)	MSCEIT	PCL-R, PPI-R	57 convicted male offenders	-Positive correlation between Fearless dominance and perceiving emotions. -Negative correlation between Self-centered impulsivity and management branch. - Positive correlation between antisocial and perceiving emotion.
(92)	MSCEIT	PPI-R	26 male inmates	-Negative relationship between EI and both the total psychopathy score and self-centered dimension.
(93)	MSCEIT	DTDD	543 students (402 females)	- Negative correlation between EI and psychopathy (women).
(94)	MSCEIT	LSRP	396 adolescents (199 female)	- Negative correlation between EI and psychopathy.
(95)	MSCEIT-YVR	PCL-YV	141 adolescents	-No significant associations between EI and psychopathy in adolescents.
(58)	MSCEIT	PCL-R	33 male inmates	-No significant associations were found between the PCL-R and MSCEIT indices.

*MSCEIT, Mayer-Salovey-Caruso Emotional Intelligence Test (4); PCL-R, The Psychopathy Checklist-Revised (35); PPI-R, The Psychopathic Personality Inventory Revised (36); SRP-III, Self-Report Psychopathy Scale-III (40); LSRP, The Levenson Self-Report Psychopathy Scale (79); DTDD, Dark Triad Dirty Dozen (86); PCL-YV, Psychopathy Checklist: Youth Version (75)*.

With respect to studies that found negative correlations between EI and psychopathy, Curci et al. (55) used PCL-R as a psychopathy scale. The results showed a negative correlation between EI and psychopathy, along with a negative correlation between trait impulsivity and the branches of strategic EI (*r* = −0.16, *p* < 0.05), which are understanding emotions and managing emotions. Vidal et al. (88) used PPI-R as a psychopathy scale and they showed a negative correlation between total MSCEIT scores and PPI-R (*r* = −0.21, *p* < 0.05), specifically, deficit in understanding (*r* = −0.19, *p* < 0.005), and managing emotions (*r* = −0.30, *p* < 0. 01). In addition, they also found that the facilitating, understanding and managing branches correlated inversely with the impulsive antisociality scale (respectively, *r* = −0.17, *p* < 0.05, *r* = −0.23, *p* < 0. 01; *r* = −0.38, *p* < 0.01).

Howe et al. (89) also used PPI-R as a psychopathy scale. They found that total EI and total psychopathy scores were negatively related (*r* = −0.40, *p* < 0.01). Besides they found the interpersonal-affective were not significantly related to EI, as long as cold heartedness and self-centered impulsivity were significantly negatively correlated with MSCEIT total scores. Moreover, cold heartedness had a negative correlation with the facilitating branch (*r* = −0.37, *p* < 0.01), and the self-centered impulsivity scale was negatively correlated with the perceiving (*r* = −0.44, *p* < 0.01), facilitating (*r* = −0.51, *p* < 0.01), and managing (*r* = −0.41, *p* < 0.01) branches.

Lishner et al. (90) used the SRP-III psychopathy scale. The results showed a negative correlation between psychopathy and its facets with MSCEIT (*r* = −0.34, *p* < 0.05). The SRP-III scale of psychopathy was also used by Visser et al. (91). The results showed a negative correlation between EI and psychopathy (*r* = −0.30, *p* < 0.05) and they found that subscales of EI are negatively correlated with the subscales of psychopathy and antisociability. Curci et al. (92) found a negative correlation between MSCEIT scores with total PPI-R (*r* = −0.56, *p* < 0.01) and the self-centered dimension of PPI-R (*r* = −0.21, *p* < 0.05). Jauk et al. (93), using the DTDD psychopathy scale, found a significant negative correlation between psychopathy and EI (*r* = −0.20, *p* < 0.05), but only in women. Finally, Zhang et al. (94) measured psychopathy with the LSRP and they found a negative relationship between EI and psychopathy (*r* = −0.23, *p* < 0.05).

In relation to the studies that found mixed correlations, Copestake et al. (57) using the PCL-R and PPI-R psychopathy scales, found that fearless dominance measured with PPI-R and the antisocial scale measured with the PCL-R were both positively correlated with the perceiving emotion branch of the MSCEIT (*r* = 0.33, *p* < 0.05). They also found that self-centered impulsivity was negatively related to the management branch of MSCEIT (*r* = −0.29, *p* < 0.05).

With respect to studies that found no relationship between EI and psychopathy, Curci et al. (58) measured psychopathy with the PCL-R and they did not find a relationship between these constructs. Kahn et al. (95) measured EI (MSCEIT-YV-R) and psychopathy (PCL-YV) in adolescents and found no relationship between these two constructs.

#### Self-report ability model

Our search identified 10 studies that employed self-report ability tests for EI (Table 2). Five of ten studies found negative correlations between psychopathy and EI, and a further two found mixed results: negative correlations for some scales and positive correlations for others. One of the studies revealed that higher EI was associated with higher psychopathy. Finally, two of the studies found no relationship between EI and psychopathy.

**Table 2:** Studies using the Self-Report ability model.

**Study**	**EI scale**	**Psychopathy scale**	**Sample (N)**	**Principal results**
(56)	WLEIS	PPI-R, PPI	92 offenders (28 female)	- No relationship between EI and psychopathy.
(96)	SEIS	LSRP	73 undergraduates (58 female)	-EI was negatively associated psychopathy.
(97)	TMMS	PCL-R	439 male inmates	-The repair and attention scores of psychopathic participants were lower than those of controls. -The clarity scores of psychopathic were higher than controls.
(57)	TMMS	PCL-R, PPI-R	57 convicted male offenders	-Positive correlation between psychopathy and clarity scale. -Positive correlation between Self-centered impulsivity and repair and clarity scale.
(98)	SSI	SRP-III	594 (438 female)	-Negative association between psychopathy and emotional sensitivity. -Positive associations between psychopathy and emotional control.
(99)	SEIS	LSRP	193 students (149 female)	-Negative association between secondary psychopathy and EI.
(100)	SEIS	LSRP	49 psychology students (39 female)	-Negative association between psychopathy and EI.
(101)	SEIS	LSRP	243 (155 female)	-Negative association between secondary psychopathy and EI.
(102)	SEIS	LSRP	234 (193 female)	-Negative association between psychopathy and EI.
(94)	SEIS	LSRP	396 adolescents (199 female)	- No relationship between EI and psychopathy in adolescents.

*WLEIS, Wong and Law Emotional Intelligence Scale (65); SEIS, Schutte's Self-Report Emotional Intelligence Scale (68); TMMS, Trait-Meta Mood Scale (6); SSI, Self-Reported Social Skills Inventory (67); PPI-R, The Psychopathic Personality Inventory Revised (36); PPI, Psychopathic Personality Inventory (80). LSRP, The Levenson Self-Report Psychopathy Scale (79); PCL-R, The Psychopathy Checklist-Revised (35); SRP-III, Self-Report Psychopathy Scale-III (40)*.

In relation to studies that found negative correlations, Grieve and Mahar (96) used the SEIS to measure EI and LSRP to assess psychopathy. They found a negative association between EI and psychopathy (*r* = −0.26, *p* < 0.05). In another study, Grieve and Mahar (100) found a negative correlation between psychopathy and EI (*r* = −0.34, *p* < 0.01). Grieve et al. (99) used the SEIS to measure EI and LSRP to assess psychopathy and found a negative correlation between EI and secondary psychopathy (*r* = −0.46, *p* < 0.001). Grieve and Panebianco (101) also used SEIS and LSRP and they found a negative correlation between EI and secondary psychopathy (*r* = −0.40, *p* < 0.001). Finally, Hyde and Grieve (102), using SEIS and LSRP, found a negative correlation between EI and primary (*r* = −0.18, *p* < 0.01) and secondary (*r* = −0.40, *p* < 0.01) psychopathy.

In relation to the studies that found mixed correlations, the TMMS EI scale and the PCL-R psychopathy scale was used in the study of Malterer et al. (97), where they found that the repair (*M* = −0.18, *SD* = 0.95) and attention (*M* = −0.15, *SD* = 1.19) scores of psychopathic participants were lower than controls controls (*M* = 0.14, *SD* = 1.04; for repair; *M* = 0.07, *SD* = 0.94 for attention), whist the clarity scores participants (*M* = 0.12, *SD* = 1.06) were higher than those of controls (*M* = 0.04, *SD* = 0.96). On the other hand, Nagler et al. (98) found a negative association between psychopathy and emotional sensitivity (*r* = −0.19, *p* < 0.001). However, they found a positive association between psychopathy and emotional control (*r* = 0.14, *p* < 0.01) and emotional manipulation (*r* = 0.71, *p* < 0.001). These researchers used SSI for EI and SRP-III for psychopathy.

When we analyzed the studies that found positive correlations, we observed that Copestake et al. (57) found a positive correlation between antisocial (PCL-R) and clarity (TMMS) (*r* = 0.32, *p* < 0.05), along with a positive correlation between Self-centered impulsivity, measured with PPI-R, and repair (*r* = 0.41, *p* < 0.01) and clarity (*r* = 0.37, *p* < 0.01) of TMMS. Finally, the total PCL-R was significantly and positively correlated with the clarity scale of TMMS (*r* = 0.36, *p* < 0.05).

Finally, we analyzed studies that did not find correlations. Ray et al. (56), using the WLEIS as EI scale and the PPI and PPI-R as psychopathic scales, did not find significant relationships (respectively, *r* = −0.28 and *r* = −0.20). Finally, Zhang et al. (94) measured psychopathy with the LSRP and found no relationship between EI and psychopathy (*r* = – 05).

#### Self-report mixed model

We identified 11 studies that used self-report mixed tests for measuring EI (Table 3). Six of eleven studies found that higher EI was linked to less psychopathy, four of the studies found mixed results: negative correlations for some scales, and others positive correlations. One of the studies found positive correlations.

**Table 3:** Studies using self-report mixed EI tests.

**Study**	**EI scale**	**Psychopathy scale**	**Sample (N)**	**Principal results**
(18)	TEIQue	LSRP	84 undergraduate students (67 female)	-Secondary Psychopathy correlated negatively with trait EI.
(103)	TEIQue	PRI, SRP	510 participants (424 female)	-Positive correlation between psychopathy and sociability factor and social awareness. - Psychopathy was negatively related to emotional perception, expression, empathy and relationship skills.
(104)	TEIQue-SF	SRP-4	100 undergraduate students (75 female)	-Psychopathy and EI were negatively related.
(105)	TEIQue	PCL-R	39 male secure psychiatric patients	-Psychopathic individual presented higher total scores of perceived EI, as well as Higher EI scores on the emotional regulation and emotional perception dimensions, in comparison with controls.
(106)	TEIQue-SF	PPI-R	150 participants (75 female)	- Higher levels of psychopathy were associated with higher levels of EI.
(107)	TEIQue	SRP	241 adults twin pairs (183 female)	- Negative correlation between EI and psychopathy.
(108)	EQ-i	PPI-R	111 male undergraduate students	-Stress management (+), intrapersonal (–), interpersonal relationship (+) and General Mood (–) predicted psychopathy.
(109)	TEIQue-SF	LSRP	369 university students (246 female)	- Negative correlation between EI and psychopathy.
(93)	TEIQue-SF	DTDD	543 students (402 female)	- Negative correlation between EI and psychopathy.
(110)	TEIQue-SF	SD3	199 undergraduates (88 female)	- Negative correlation between EI and psychopathy.
(111)	EQi	PPI-SF	1257 (70% Female)	-PPI FD was generally positively associated with self-reported EI. -PPI SCI negatively associated with EI. - PPI C was positively but weakly correlated with EQi Adaptability and Stress Management.

*TEIQue, Trait Emotional Intelligence Questionnaire (10); EQ-i, Bar-On EQ-I (72); TEIQue-SF, Trait Emotional Intelligence Questionnaire-Short Form (71); LSRP, The Levenson Self-Report Psychopathy Scale (79); PRI, Neo Psychopathy Resemblance Index (81); SRP, Self-Report Psychopathy Scale (82); PCL-R, The Psychopathy Checklist-Revised (35); SRP-4, Self-Report Psychopathy Scale (86); PPI-R, The Psychopathic Personality Inventory Revised (36); SRP, Self-Report Psychopathy Scale (82); SD3, Short Dark Triad (87)*.

In relation to studies that found negative correlations between EI and psychopathy, Ali et al. (18) used the TEIQue EI instrument and the LSRP to measure psychopathy. They found a negative correlation between secondary psychopathy and EI (*r* = −0.41, *p* < 0.01). Petrides et al. (107) found a negative correlation between EI and psychopathy (*r* = −21, *p* < 0.01) when they used TEIQue to measure EI and the SRP to measure psychopathy. Porter et al. (104) also found negative correlations between EI, measured with the TEIQue-SF, and psychopathy (*r* = −0.29, *p* < 0.05), measured with the SRP-4. Austin et al. (109) used TEIQue-SF to measure EI and LSRP to measure psychopathy, and they found a negative correlation between EI and primary (*r* = −0.25, *p* < 0.01) and secondary (*r* = −0.57, *p* < 0.001) psychopathy. Jauk et al. (93) used the TEIQue-SF EI instrument and the DTDD to measure psychopathy and found a negative correlation between EI and psychopathy (*r* = −0.20, *p* < 0.001). Finally, Plouffe et al. (110) used TEIQue-SF to measure EI and SD3 to measure psychopathy, and they also found a negative correlation between EI and psychopathy (*r* = −0.23, *p* < 0.01).

In relation to articles that found positive correlations, Pham et al. (105) also used the TEIQue EI test and the PCL-R psychopathy test. Their study showed that psychopaths presented higher EI total scores and a higher degree of regulation and perception than controls [*F*_(1, 37)_ = 4.20, *p* < 0.05].

Finally, in relation to the studies that found mixed correlations, Tapscott et al. (103) used TEIQue as a measure of EI and the PRI, and SRP as psychopathy scales. Their research found that PRI positively correlated with the social awareness (*r* = 0.32, *p* < 0.001), and sociability factors (*r* = 0.49, *p* < 0.001); however, SRP was negatively related to emotional perception (*r* = −0.21, *p* < 0.01), expression (*r* = −0.27, *p* < 0.01), empathy (*r* = −0.30, *p* < 0.001), and relationship skills (*r* = −0.42, *p* < 0.001). Sacco et al. (106) decided to use TEIQue-SF for EI and PPI-R as psychopathy scale. Their research showed that higher levels of psychopathy were associated with higher levels of EI (*r* = −0.57, *p* < 0.001). Fix and Fix (108) used EQI-C as a measure of EI and PPI-R for psychopathy. They found that psychopathic traits were predicted through Stress Management [β = 0.29, *t*_(110)_ = 2.90, *p* = 0.005] and General Mood [β = −0.30, *t*_(110)_ = −3.15, *p* = 0.002] and also Interpersonal Relationships was a significant predictor of psychopathy [β = 0.32, *t*_(110)_ = 3.79, *p* = 0.001]. Finally, Watts et al. (111) employed the EQi for EI and PPI-SF as psychopathy scale, and they found that PPI FD was generally positively associated with EI (*r* = 0.26, *p* < 0.001). PPI C was positively but weakly correlated with EQi Adaptability (*r* = 0.08, *p* < 0.01) and Stress Management (*r* = 0.12, *p* < 0.005), but PPI SCI was negatively correlated with EI (*r* = −0.34, *p* < 0.001).

In this section, there are no studies that found the absence of a relationship between psychopathy and EI.

## Discussion

**Figure 2:**
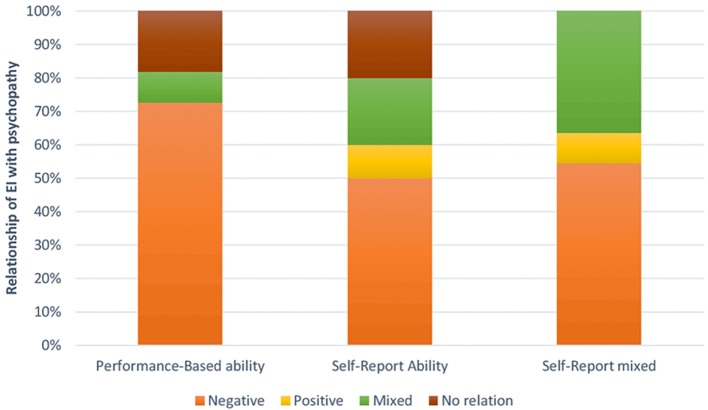
Relationship between psychopathy and EI measured through self-report ability tests, self-report mixed tests, or performance tests in the total sample.

If we analyse the results of the total sample (Figure 2), we can see that if we base our analysis on studies employing the performance-based ability model, we find that 72.72% of the articles show a negative correlation between EI and psychopathy. While only 50% of articles found this correlation when using the self-report ability model, and, when adopting the self-report mixed model, 54.54% of the studies found this relationship.

Moreover, we found that if we based our analysis on the performance-based ability model, we did not find any article revealing a positive correlation between EI and psychopathy; however, for self-report ability model, we found 10% of studies revealed positive correlations and 9.09% in self-report mixed model. Further, we only found that 9.09% of articles based on the performance-based ability model had mixed results, whereas 20 and 36.36% of the studies yielded mixed results when using the self-report ability and mixed models, respectively. Finally, two studies (18.18%) based on the performance-based ability model found no relationship between EI and psychopathy.

If we focus on the results regarding the most relevant psychopathy questionnaires and the correlations with the most consistent EI model (the Performance-Based Ability Model), it can be seen that Factor 2 of the PCL (social deviance) is negatively and significantly related to EI in all the studies included. This factor is not specific to psychopathy and is characterized by general impulsivity, irresponsibility, and past criminal and antisocial behavior.

Secondly, in relation to the PPI scale, the most consistent results with the Performance-Based ability model are those with Factor 2 (Self-Centered Impulsivity) and only in the adult population (neutral results were found in adolescents). The majority of these studies found that higher scores on Factor 2 of PPI, which contains the impulsive-behavioral features of psychopathy, were associated with lower EI.

Finally, if we focus on the models that use self-reports to measure EI, it is more difficult to find patterns in relation to the questionnaires that are used to measure psychopathy, since these results are more inconsistent.

### Clinical/inmate sample

Of the 29 articles in our systematic review, seven used the clinical/inmate sample where EI is measured eight times. Again, we categorized these results according to the type of EI model employed. We assigned participants to the clinical/inmate sample if they had been in prison, committed crimes, or had been admitted to a psychiatric center.

#### Performance-based ability model

Ermer et al. (55) used a total of 374 male inmates. In this study MSCEIT is used to measure EI and PCL-R to measure psychopathy. As discussed in the general results section, this research highlights the negative correlation between the total psychopathy score and the strategic branch (understanding and managing emotions) of MSCEIT. In addition, we also found that the impulsivity trait of psychopathy correlated negatively with the same two branches of strategic EI.

Secondly, Curci et al. (92) used a sample of 26 male inmates. They found a negative correlation between the total MSCEIT scores and both the total PPI-R scores and the self-centered dimension of the PPI-R.

Thirdly, in Copestake et al. (57), the sample was composed of 57 convicted male offenders. They found a positive correlation between fearless dominance and the antisocial scale with the perceiving emotion branch, while they found a negative correlation between Self-centered impulsivity and the management branch of MSCEIT.

Finally, in the study by Curci et al. (58), the sample was composed of 33 male inmates. The latter authors used MSCEIT as the EI scale and PCL-R as the psychopathy scale and found no evidence of a relationship between EI and psychopathy.

#### Self-report ability model

We found three articles in our review that used the Self-report ability model with the clinical/inmate sample. Firstly, Ray et al. (56) used a sample of 92 offenders (28 female) and adopted the WLEIS as a measure of EI and PPI-R and PPI as measures of psychopathy. They found a negative but non-significant correlation between EI and psychopathy.

Second, the sample used by Malterer et al. (97) consisted of 439 male inmates and they employed the TMMS to measure EI, and the PCL-R to measure psychopathy. They found an interaction between psychopathy and TMMS scores on repair, as long as scores on psychopathic care were lower than those of controls. However, clarity scores were higher in psychopathic subjects than controls.

Finally, the sample used by Copestake et al. (57) was composed of 57 convicted male offenders. The TMMS was used to measure EI, and for psychopathy they used the PCL-R and the PPI-R. They found a positive correlation between Self-Centered impulsivity, repair, and clarity scales of EI and a positive correlation between total psychopathy score and the clarity scale.

#### Self-report mixed model

We only found one article using the Self-Report Mixed Model together with a clinical/inmate sample (105). In this case, participants were 39 male patients of a secure psychiatric unit. The measures used were the TEIQue for EI and the PCL-R for psychopathy. They found a positive correlation between psychopathy and EI, specifically a positive correlation between psychopathy and the perception scale of EI. In addition, they found that psychopaths presented greater emotional regulation and perception than control participants.

#### Discussion

**Figure 3:**
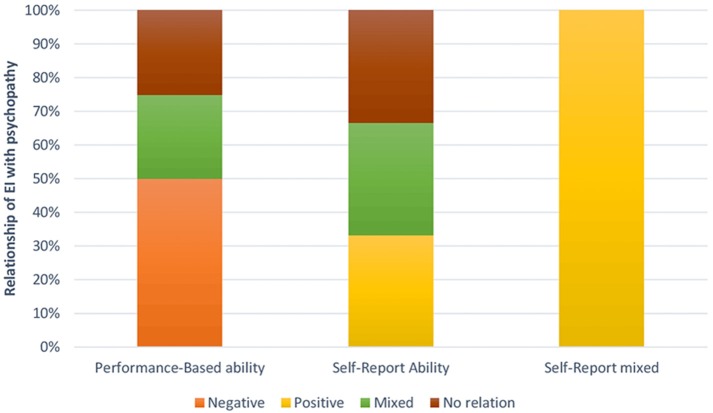
Relationship between psychopathy and EI measured through self-report ability tests, self-report mixed tests, or performance tests in a clinical/ inmate sample.

If we analyse the results of the clinical/inmate sample displayed in Figure 3, we can observe that when focusing on the performance-based ability model, in the four articles found in this section two was a negative correlation between EI and psychopathy, that is to say 50%, compared with the 0% found when the analysis was based on the self-report ability model and self-report mixed model. Further, with respect to positive correlations between EI and psychopathy, we can see that for the performance-based ability model there was 0% correlations compared with 33.3% when basing the analysis on the self-report ability model, and 100% for the self- report mixed model. Finally, there was one set of mixed results in one of the articles that used the self-report ability model along with another set of mixed results in one article that used the ability model.

## General discussion

In the present study, we carried out a systematic review examining the literature where the relationship between EI (measured through any of the three main models) and psychopathy appears in the total sample and clinical/inmate sample. We found 29 suitable studies that used 13 different scales. Psychopathy was measured with 11 different scales. When we only consider articles that use the clinical/inmate sample, we found just seven studies.

With respect to the total sample, we can observe that when the studies used the performance-based ability model, the majority of participants (72.72%) who presented deficits in EI obtained a high score in psychopathy, compared with studies based on self-reports where there was a small percentage of high psychopathy participants who exhibited EI deficits (50% in the studies using the self-report ability model and 54.54% in those using the self-report mixed model). These results are consistent with previous studies where it has been demonstrated that the performance-based ability model has a greater predictive capacity for emotional cognitive processes than the self-report models (60, 112). In addition, these results are expected, given that the literature shows that a person with a high degree of psychopathy presents deficits in emotional aspects (24, 27). In particular, in studies based on the Performance-Based model, two articles found the absence of a relationship between EI and psychopathy. It is important to note the findings of the study by Copestake et al. (57). In their work, they found a negative correlation between psychopathy and the managing branch of the MSCEIT, but a positive relationship between perception of emotions and psychopathy, that is, a higher score on the psychopathy scale was related to greater emotional perception. However, there are some studies suggesting that the excessive perception of emotions could be a counterproductive trait (113, 114), and thus might not always be regarded as positive.

It appears, therefore, that the results of studies adopting the performance-based ability model are much more consistent in predicting psychopathy than those based on self-report instruments. We found numerous inconsistencies between the findings of studies that used self-reports. In particular, there were studies that showed that higher EI participants had less psychopathy, while others showed that lower EI participants had lower psychopathy, and some found mixed results. This is not surprising given that these instruments focus on the subjective perception of individuals, and such perceptions that do not always match their actual abilities (115, 116). Therefore, it is difficult to establish such instruments as adequate predictors of levels of psychopathy.

If we focus on the main instruments of psychopathy, we can conclude, in general terms, that when using the performance-based ability model of EI, this is more related to Factor 2 of the PCL instrument (the social deviance), which is characterized by impulsive lifestyle and antisocial traits [e.g., (55)] and also to Factor 2 of PPI (Self-Centered impulsive) based on impulsive-behavioral features [e.g., (57)]. In models that use self-reports to measure EI, it is more complicated to find a discernible pattern since the results are inconsistent.

With the clinical/inmate sample, we found results similar to the total sample, when studies are based on the performance-based model. In particular, greater EI is related to lower psychopathic symptomatology, while the self-report literature presents rather fuzzy results, which include negative and positive correlations, as well as mixed results with regard to the relationship between EI and psychopathy. However, these results cannot be conclusive since the sample used here was relatively small, with only four performance-based ability model studies, three self-Report ability studies, and one with the Self-Report mixed model. Therefore, future research should aim at studying the relationship between EI and psychopathy using the clinical/inmate sample, given the importance of the relationship between EI and the crimes committed by individuals in this sample (35, 52).

One limitation is that the studies included in the review use correlations and are therefore unable to predict causality. Moreover, there are a great variety of instruments to measure psychopathy, specifically 11 different instruments, which do not cover the same scales. Finally, an important limitation is that we were not able to conduct a meta-analysis (although this could have provided more information about the results) due to the different characteristics of the questionnaires used in the studies. For instance, unlike the MSCEIT (Performance-Based Ability Model), the TMMS (Self-report Ability Model) instrument does not have a global scoring system, which makes comparisons difficult.

This study has clinical implications, which we also suggest could form the basis of potentially new areas of research. First, future research should aim to empirically show the possibility of using EI, measured through performance-based ability model instruments, as a method of evaluation in the psychopathic population. Evaluating EI through performance-based ability models could become another criterion with which to improve the diagnoses in this population. Further, given the shortcomings of this population, EI training may be of benefit in alleviating emotional deficits, and could also be employed as a preventive intervention in children and adolescents (51, 54) to reduce the levels of aggression that characterize part of this population (48). It would also be interesting to study the possible benefit of training EI in prisons to reduce violent behavior. Further, it would be useful to examine whether an improvement in aspects related to EI such as empathy, or a decrease in the manipulation of others could help people with psychopathic traits and social problems to adapt better to society (27, 44). Finally, in all the aforementioned applications, personality variables such as anxious or depressive traits should be taken into account in order to adapt the evaluation, and to train EI in the most appropriate way for each individual.

In conclusion, this systematic review helps us to better understand the relationship between psychopathy and EI. If we base our analysis on studies using the performance-based ability model—which is the most consistent model in predicting the psychopathic traits—we find that in most studies there is a negative relationship between psychopathy and EI, a finding that did not emerge when reviewing the literature using self-report measures. This leads us to a series of clinical implications, such as the possible evaluation, prevention, and treatment of psychopathy through the study of EI.

## Author contributions

RG-L wrote the paper, drafting the work critically for important intellectual content. MG-C substantial contributions to the design of the work and interpretation of data for the work, drafting the work critically for important intellectual content. RC and PF-B substantial contributions to the conception of the work and interpretation of data for the work, revising the work critically for important intellectual content. AM revising the work critically for important intellectual content. All authors final approval of the version to be published and Agreement to be accountable for all aspects of the work in ensuring that questions related to the accuracy or integrity of any part of the work are appropriately investigated and resolved.

### Conflict of interest statement

The authors declare that the research was conducted in the absence of any commercial or financial relationships that could be construed as a potential conflict of interest.
